# Development, Pilot Study, and Psychometric Analysis of the AHRQ Surveys on Patient Safety Culture™ (SOPS^®^) Workplace Safety Supplemental Items for Hospitals

**DOI:** 10.3390/ijerph19116815

**Published:** 2022-06-02

**Authors:** Katarzyna Zebrak, Naomi Yount, Joann Sorra, Theresa Famolaro, Laura Gray, Deborah Carpenter, Andrew Caporaso

**Affiliations:** Westat, Rockville, MD 20850, USA; naomiyount@westat.com (N.Y.); joannsorra@westat.com (J.S.); theresafamolaro@westat.com (T.F.); lauragray@westat.com (L.G.); andrewcaporaso@westat.com (A.C.)

**Keywords:** healthcare, health care, workplace safety, workforce safety, patient safety, safety culture, organizational culture, survey, hospital, psychometric analysis

## Abstract

Workplace safety is critical for advancing patient safety and eliminating harm to both the healthcare workforce and patients. The purpose of this study was to develop and test survey items that can be used in conjunction with the Agency for Healthcare Research and Quality (AHRQ) Surveys on Patient Safety Culture™ (SOPS^®^) Hospital Survey to assess how the organizational culture in hospitals supports workplace safety for providers and staff. After conducting a literature review and background interviews with workplace safety experts, we identified key areas of workplace safety culture (workplace hazards, moving/transferring/lifting patients, workplace aggression, supervisor/management support for workplace safety, workplace safety reporting, and work stress/burnout) and drafted survey items to assess these areas. Survey items were cognitively tested and pilot tested with the SOPS Hospital Survey 2.0 among providers and staff in 28 U.S. hospitals. We conducted psychometric analysis on data from 6684 respondents. Confirmatory factor analysis results (item factor loadings and model fit indices), internal consistency reliability, and site-level reliability were acceptable for the 16 survey items grouped into 6 composite measures. Most composite measures were significantly correlated with each other and with the overall rating on workplace safety, demonstrating conceptual convergence among survey measures. Hospitals and researchers can use the Workplace Safety Supplemental items to assess the dimensions of organizational culture that support provider and staff safety and to identify both strengths and areas for improvement.

## 1. Introduction

In 2020, the Institute for Healthcare Improvement’s (IHI) National Steering Committee for Patient Safety (NSC) released a National Action Plan to Advance Patient Safety in the United States (U.S.) that identified four foundational areas for patient safety: Culture, Leadership and Governance; Patient and Family Engagement; Learning Systems; and Workforce Safety [1]. The report identified workforce safety—the physical and psychological well-being and safety of the healthcare workforce—as a necessary precondition to advancing patient safety and highlighted the need for a systems approach to eliminate harm to both patients and the workforce. Further, IHI’s National Action Plan identified several priority areas for workforce safety assessment and program implementation, including safe patient handling as well as prevention of exposures (e.g., to pathogens and chemicals), sharps and needlestick injuries, slips/trips/falls, and workplace violence [1].

U.S. healthcare workers experience over 582,000 injuries each year [2]. Based on the 2020 data from the Bureau of Labor Statistics (BLS), the overexertion injury rate for hospital workers is more than twice the national average of U.S. full-time workers [3]. The most important risk factor for these injuries is the manual lifting, moving, and repositioning of patients [4]. Despite progress resulting from safe patient handling and mobility legislation, U.S. acute care facilities continue to underuse lifts to safely mobilize patients [5].

According to the Centers for Disease Control and Prevention (CDC) [6], approximately 385,000 needlestick and sharps injuries occur annually among hospital employees. However, research suggests that those injuries are frequently underreported [7,8], and therefore the actual number is likely much higher. Effective organizational policies, procedures, and programs can help to prevent sharps injuries and facilitate reporting [6]. In addition to needlestick and sharps injuries, slips, trips, and falls represent another common hazard in healthcare settings, resulting in injury and loss of workdays [9].

Workplace violence and aggression, including both verbal and physical acts, are ubiquitous and underreported in healthcare settings, with healthcare workers being four times more likely to be victimized than workers in private industry [10,11]. Aggression toward healthcare workers can come from multiple sources, including patients, visitors, and coworkers, and may result in negative physical, psychological, social, and emotional sequelae [12].

Underreporting of workplace concerns, hazards, and incidents is common in healthcare and occurs for various reasons, including a fear of negative consequences, repercussions, or blame; perceived burden or difficulty in reporting; time constraints; and lack of awareness of the reporting systems [13,14,15]. Healthcare organizations can encourage reporting by having clear policies and reporting mechanisms, supporting non-punitive attitudes, and protecting worker confidentiality [16].

The COVID-19 pandemic has further compromised the safety of healthcare workers. Shortages of appropriate personal protective equipment (PPE) in the early phases of the pandemic increased the risk of provider and staff exposure to SARS-CoV-2 and exacerbated their fears of both becoming infected and infecting co-workers and family members [17,18]. The physical and psychological well-being of healthcare workers was further strained by increased patient loads and staffing shortages, all of which significantly contribute to work stress and burnout in the healthcare workforce [19,20]. Thus, the COVID-19 pandemic underscored the need for ongoing organizational support to protect the physical, mental, and emotional safety and well-being of the healthcare workforce [21].

In response to increased concern about the safety of healthcare workers during the COVID-19 pandemic and recognizing the importance of workplace safety in ensuring patient safety, Westat was contracted by the Agency for Healthcare Research and Quality (AHRQ) to develop survey items focusing on workplace safety for providers and staff in the hospital setting. The items were designed and tested as a supplement to the AHRQ Surveys on Patient Safety Culture™ (SOPS^®^) Hospital Survey 2.0 that assesses the extent to which organizational culture in hospitals supports patient safety [22]. Organizational culture refers to the beliefs, values, and norms shared by clinicians and staff within healthcare organizations which influence their actions and behaviors [23]. Similar to the SOPS Hospital Survey, the supplemental items would enable hospitals to assess the extent to which their organizational culture supports the safety of providers and staff.

In healthcare, a comprehensive safety culture includes both patient and workplace safety [24]. Since workplace safety culture and patient safety culture are related [25], hospitals can use these supplemental items to obtain provider and staff perspectives on workplace safety, with a goal of identifying strengths and areas of improvement related to both workplace and patient safety. Herein we present information on the survey development process, pilot study data collection, and results from psychometric analysis of the supplemental items.

## 2. Materials and Methods

### 2.1. Supplemental Item Development

**Figure 1:**
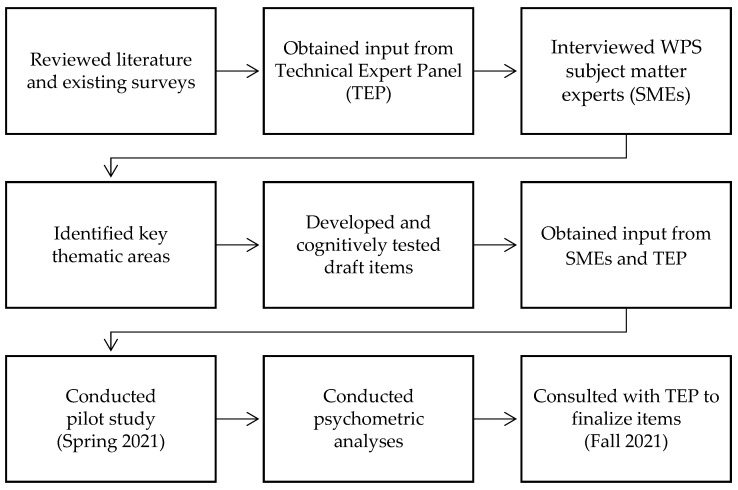
Workplace safety supplemental item development process.

Figure 1 shows the development process for the Workplace Safety Supplemental Items. The supplemental items were developed using an iterative process starting with a non-systematic review of published and grey literature in various areas of workplace safety, including slips, trips, and falls; sharps injuries; psychological safety and well-being; safe patient handling; violence and aggression; workplace safety reporting; and work stress/burnout. We also reviewed extant literature on organizational culture factors that affect the workplace safety of providers and staff, in addition to any existing survey measures on workplace safety. To supplement the results of literature searches and better understand the key elements of workplace safety in healthcare settings, we conducted in-depth interviews with eight workplace safety experts selected based on their content expertise from diverse settings, including universities, healthcare systems, and professional healthcare associations.

After synthesizing the information from these sources, we identified key dimensions of workplace safety that were widely applicable to hospital staff, relevant to organizational culture in hospitals, and could be assessed using closed-ended, self-reported survey items. Next, we drafted survey items to assess those key dimensions. As the funding agency, AHRQ provided ongoing input and feedback throughout survey development. In addition, an 18-member Technical Expert Panel (TEP), which included several workplace safety experts, provided feedback on survey dimensions and items at several stages of survey development, starting with the first draft. Based on feedback from the TEP, we revised the initial draft dimensions and survey items in preparation for cognitive testing.

To assess item comprehension, relevance, and ease of responding, we cognitively tested the draft supplemental items in three rounds of cognitive interviews with 15 providers and staff in English and one round with five providers and staff in Spanish. Participants included physicians, nurses, and administrative/support staff in order to assess the applicability of the items across different positions in hospitals. After each round of cognitive testing, we revised, added, or dropped items based on the results of the cognitive interviews or continued testing them, as appropriate. Members of the TEP provided feedback on the draft items after each round before we tested the revised versions.

Although we identified important areas of hospital workplace safety through our literature review, there were some areas that were difficult to assess with survey items. During cognitive interviews, we learned that sharps injuries did not apply to all respondents. Additionally, when a staff member experienced a sharps injury, it was often attributed to staff error rather than facility policies, which was overly punitive for staff and not adequately assessing organizational culture. Another area that was difficult to assess was slips, trips, and falls, as staff were thinking about both patients and workers when answering those questions. Therefore, we did not include questions about those areas in the pilot survey. After cognitive testing and TEP input, the survey items were finalized for the pilot study.

### 2.2. Measures

The pilot study supplemental items consisted of 31 survey items assessing seven a priori dimensions or composite measures of workplace safety in hospitals (see Table 1). Composite measures are groups of two or more survey items that measure the same conceptual domain. Multiple survey items were developed to assess each area more comprehensively, maximize content validity and reliability of measurement, and provide more specific and actionable information to hospitals.

In addition to these composite measures, the survey also included one single-item measure, *Work Stress/Burnout*, and one *Overall Rating on Workplace Safety for Providers and Staff*, and several background questions on respondent characteristics.

Most questions were asked using five-point Likert-type scales with response options from *Strongly disagree* to *Strongly agree* or *Never* to *Always* and included a *Does Not Apply or Don’t Know* as the sixth response option. Eight of the *Workplace Safety and Reporting* items included a set of screener questions asking respondents if they noticed or experienced a safety issue in the past 12 months (e.g., unsafe working conditions, aggression) and, if the response was “Yes”, how often they reported the safety issue. The *Work Stress/Burnout* item was adapted from an item on the Mini-Z instrument (we modified the first response option from *I enjoy my work. I have no symptoms of burnout* to *I have no symptoms of burnout*, as we found during cognitive testing that some respondents indicated enjoying their work but also reported experiencing some symptoms of burnout) [26]. Providers and staff who indicated feeling burned out (i.e., *The symptoms of burnout that I am experiencing won’t go away. I think about work frustrations a lot*, or *I feel completely burned out. I am at the point where I may need to seek help*) were encouraged to consider seeking assistance (e.g., from their insurance provider or employee assistance plan [EAP]). At the beginning of the Workplace Aggression section, we provided definitions and examples of physical and verbal aggression.

### 2.3. Pilot Study

We conducted a pilot study in 28 hospitals across 16 states in the U.S. from May to June 2021. The purpose of the pilot study was to obtain data for psychometric analyses to examine the reliability and construct validity of the items, with the goal of retaining the best performing items. Hospitals were recruited from AHRQ SOPS email listserv subscribers, SOPS Hospital Survey users, webinars, and through outreach to hospital stakeholder organizations. Hospitals were selected to vary by several characteristics (e.g., bed size, region, ownership, teaching status), but were not statistically representative of all U.S. hospitals. The workplace safety items were added toward the end of the SOPS Hospital Survey 2.0 [22] (just before the background questions) and administered by Westat as a web-based survey to a census of all providers and staff in the hospitals. Each provider and staff member received an email with a unique survey link. Data collection procedures were the same across all hospitals.

### 2.4. Analyses

Psychometric analyses included: (1) item analysis to examine the variability of responses and percentages of missing data, (2) internal consistency reliability, (3) confirmatory factor analysis, (4) site-level reliability, and (5) site-level percent positive scores and correlations among composite measures and items. Each of these analyses is described in more detail below.

#### 2.4.1. Item Analysis

We first examined item frequencies at the respondent level to review response variability and identify items with high percentages of missing data or *Does Not Apply/Don’t Know* (*DNA/DK*) responses. Items with little response variability may not be helpful in differentiating higher-scoring from lower-scoring hospitals. Accordingly, any items with more than 90 percent of respondents responding positively (e.g., those answering *Strongly agree/Agree* or *Always/Most of the time* for positively worded items, and *Strongly disagree/Disagree* for negatively worded items) were considered to have low variability. If more than 30% of respondents left an item missing or answered *DNA/DK,* the item was considered for removal because it may not be relevant to a large proportion of respondents. However, we did not rely solely on items flagged based on item analysis to determine which items to drop. We also examined results from other psychometric analyses and TEP feedback, weighing the importance and relevance of item content.

#### 2.4.2. Internal Consistency Reliability

Internal consistency reliability indicates how consistently respondents answer items within a composite measure by assessing how closely those items are related or correlated. Internal consistency reliability was assessed for each composite measure using Cronbach’s alpha (α). Cronbach’s alpha ranges from 0.00 to 1.00, with higher values indicating greater internal consistency. The minimum criterion for acceptable reliability is an alpha of 0.70 [27].

#### 2.4.3. Confirmatory Factor Analysis (CFA)

The purpose of CFA is to confirm a particular pattern of relationships among survey items predicated on past research and theory by assessing how well a proposed factor structure fits the data [28]. A CFA was conducted on the final proposed composite measures and their associated items. Full-information maximum likelihood estimation was employed to address missingness resulting from either nonresponse or those answering *Does not apply/Don’t Know* [29,30].

We examined standardized factor loadings for each item based on its respective composite measure. Factor loadings above 0.40 indicate that the item’s relationship to the a priori composite measure is acceptable [31]. Several model fit indices were also examined to determine how well the hypothesized factor structure fits the data (Table 2). The chi-square goodness-of-fit statistic assesses the discrepancy between the sample covariance matrix and the model-specified covariance matrix. Lower and non-significant values indicate good model fit. Since chi-square tends to be larger and statistically significant in larger samples, we examined the chi-square divided by its degrees of freedom, which is less sensitive to sample size. Values less than 5.00 indicate good model fit [32]. The comparative fit index (CFI) compares the existing model fit with a null model that assumes the factors in the model are uncorrelated. A CFI value of 0.95 or above indicates adequate model fit [33]. The root mean square error of approximation (RMSEA) is a parsimony-adjusted index that favors the simplest model possible [34]. A value less than 0.06 for RMSEA indicates good model fit [33]. The standardized root mean square residual (SRMR) is the standardized difference between the observed covariance and predicted covariance. A value of less than 0.08 for the SRMR indicates good model fit [35].

#### 2.4.4. Site-Level Reliability Analysis

We computed site-level reliability to examine the variability of item and composite measure scores among hospitals. Site-level reliability indicates the extent to which responses given by providers and staff within the same hospital are more similar to each other than they are to responses given by providers and staff from other hospitals. In other words, site-level reliability helps to assess how well a measure differentiates hospitals. It does so by comparing between-hospital variability to within-hospital variability of items. Site-level reliability was computed using the following formula [36]:

$$\mathrm{Site}-\mathrm{level}\;\mathrm{Reliability}=\frac{\mathrm{between}\;\mathrm{hospital}\;\mathrm{variance}}{\mathrm{between}\;\mathrm{hospital}\;\mathrm{variance}+\frac{\mathrm{within}\;\mathrm{hospital}\;\mathrm{variance}}{n}}$$

where n is the number of respondents in a given hospital. Site-level reliability for each measure was first computed for individual hospitals and then averaged across hospitals. This approach helps to take into consideration the differences in the number of respondents in each hospital. Similar to internal consistency reliability, values of 0.70 or higher are considered acceptable for site-level reliability [27].

#### 2.4.5. Hospital-Level Percent Positive Scores and Correlations

We calculated hospital-level percent positive scores as the percentage of respondents within a site who answered positively (% *Strongly agree/Agree* or *Always/Most of the time* for positively worded items, and % *Strongly disagree/Disagree* for negatively worded items) for each item. These site-level percent positive scores for the items within each composite measure were equally weighted and averaged to compute site-level composite measure scores. Percent positive scores can range from 0 to 100. We examined Spearman’s rank order correlations among the composite measures, single-item measures, and the overall rating. The Workplace Safety measures should be intercorrelated, as they are all designed to assess aspects of culture focusing on workplace safety. Moderate to moderately high correlations typically indicate a correspondence or convergence among similar concepts.

## 3. Results

Across the 28 hospitals that participated in the pilot study, 7037 providers and staff out of 19,979 responded, for an overall response rate of 35%. On average, there were 251 respondents per hospital (ranging from 21 to 1373). Of the 7037 respondents, 353 did not answer any Workplace Safety items and thus were excluded from the final analysis dataset, which resulted in a final analytic sample of 6684 respondents.

### 3.1. Pilot Hospital Characteristics

As shown in Table 3, approximately one-third of the pilot hospitals had 50–99 beds (32%), and most were teaching hospitals (64%). Hospitals varied in ownership: government (federal and non-federal) (39%), non-government not for profit (39%), and investor owned (for profit) (21%).

### 3.2. Characteristics of the Respondents

More than one-third (35%) of respondents were nursing staff, followed by support staff (20%); other clinical staff (18%); other positions (13%); supervisor, manager, clinical leader, or senior leader (12%); and physician, physician assistant, or resident (2%) (Table 4). The largest percentage of respondents worked primarily in the patient care unit (28%) and the smallest in surgical services (4%).

### 3.3. Item Analysis

We examined the variability of responses to survey items at the individual or respondent level. Table 5 shows the average percent positive and percent missing and *Does not apply/Don’t know* (*DNA/DK*) responses for items in the a priori composite measures, as well as one *Workplace Safety and Reporting* item and one *Overall Rating on Workplace Safety for Providers and Staff*. While there were also eight other items measuring *Workplace Safety and Reporting*, these items are not shown in Table 5 because they were dropped based on their complexity, length, and some redundancy in content with items in other sections.

Four items had low variability: three items in *Protection from Workplace Hazards* (range, 91% to 92%) and one workplace aggression item (“In this unit, there is a problem with providers or staff being physically aggressive toward other providers or staff” (91% of respondents Strongly Disagreed/Disagreed)). The remaining items had acceptable variability, with percent positive scores below 90% (range 45% to 89%). We did not find any items with a high percentage of missing responses; all items had < 5% missing responses (range <1% to 4%).

The percentages of *DNA/DK* responses ranged from 5% to 43%. Two items in *Moving, Transferring, or Lifting Patients* had high percentages of *DNA/DK*: “Equipment or assistive devices are available when needed to help move, transfer, or lift patients in this unit” (42%) and “In this unit, staff use equipment or assistive devices when needed to help move, transfer, or lift patients, even if it takes more time” (43%). When these items were further investigated, the percent *of DNA/DK* was not as high (<30%) for the majority of positions in which staff are likely to engage in moving, transferring, or lifting patients, such as registered nurses; patient care aides, hospital aides and nursing assistants; physical, occupational, or speech therapists; respiratory therapists; and transporters.

Table 6 shows responses for the item assessing *Work Stress/Burnout*. While 69% of respondents indicated having no symptoms of burnout, 32% reported some symptoms of burnout, including 3% who reported feeling completely burned out. This item had 3% missing responses.

#### 3.3.1. Internal Consistency Reliability Analysis

All a priori composite measures had initial internal consistency reliability at or above criterion (α ≥ 0.70). Cronbach’s alpha ranged from 0.70 on *Addressing Workplace Aggression from Providers or Staff* to 0.96 on *Hospital Management Support for Workplace Safety*.

#### 3.3.2. TEP Review and Input

We presented the initial analysis results to the Workplace Safety TEP to obtain their input on whether to retain or drop items. To shorten the survey, we also asked the TEP to identify other items to drop based on content relevance. After TEP feedback, five survey items were dropped: one item each from *Protection from Workplace Hazards*, *Addressing Workplace Aggression from Patients or Visitors*, and *Supervisor, Manager, or Clinical Leader Support for Workplace Safety*, and two items from *Addressing Workplace Aggression from Providers or Staff*. Details about which items were dropped and reasons for dropping these items are provided in Table S1.

#### 3.3.3. Confirmatory Factor Analysis (CFA)

When we conducted a confirmatory factor analysis (CFA) on the retained survey items, the resulting model showed poor fit to the data based on all fit indices. In addition, standardized factor loadings for items in *Addressing Workplace Aggression from Patients or Visitors* were lower relative to factor loadings obtained for other composite measures. Thus, we conducted an exploratory factor analysis on the four remaining items in that measure using iterated principal axis factors as the method of extraction, with varimax (orthogonal) rotation. Results supported a two-factor solution rather than one overall factor. The first factor had two items focusing on physical and verbal aggression from patients or visitors (*Addressing Workplace Aggression from Patient or Visitors*). The second factor had two items focusing on effective policies and procedures and training on how to deescalate or calm down aggressive behavior from patients or visitors (*Workplace Aggression Policies, Procedures, and Training*). Since only one aggression item remained in the *Addressing Workplace Aggression from Providers and Staff* a priori composite measure, “In this unit, there is a problem with providers or staff being verbally aggressive toward other providers or staff”, this composite measure became a single-item measure (*Addressing Verbal Aggression from Providers or Staff*).

To test the fit of the six proposed composite measures to the data, we reran the CFA. Table 7 displays standardized factor loadings for each of the Hospital Workplace Safety Supplemental Items on their respective composite measures. All factor loadings were statistically significant (*p* < 0.05) with magnitudes greater than 0.40, indicating that the items adequately loaded on the composite measures. The factor loadings ranged from 0.59 to 0.95, with an average of 0.85.

Three of the four goodness-of-fit indices for the six-factor model satisfied the criteria for acceptable fit of the model to the data (Table 8). Specifically, the CFI was 0.98 (criterion is ≥0.95), the SRMR was 0.05 (criterion is <0.08), and the RMSEA was 0.04 (criterion is <0.06). The chi-square value divided by the degrees for freedom was 13.5, which is above the criterion of <5.00.

After confirming the measurement structure, we re-calculated Cronbach’s alpha for the six composite measures. As presented in Table 7, the reliability estimate for *Workplace Aggression Policies, Procedures, and Training* was 0.67, slightly under the criterion of 0.70. The remaining alpha coefficients ranged from 0.83 for *Moving, Transferring, or Lifting Patients* to 0.96 for *Hospital Management Support for Workplace Safety*. Dropping one item “In this unit, enough staff are available when needed to help move, transfer, or lift patients” from the *Moving, Transferring, or Lifting Patients* would have increased the alpha from 0.83 to 0.88. However, upon careful review, we determined that the conceptual importance of this item outweighed any increase in reliability that would result from its removal. Dropping any other items from their respective composite measures would not have resulted in increases in reliability.

#### 3.3.4. Site-Level Reliability

Table 7 also shows that site-level reliability estimates ranged from 0.76 to 0.86 for the composite measures scores and ranged from 0.62 to 0.86 for the single item scores. Only 2 of the 20 items had site-level reliability under the criterion of 0.70: “In this unit, there are effective policies and procedures to keep providers and staff safe from aggressive patients or visitors” (reliability = 0.69) and *Work Stress/Burnout* (reliability = 0.62).

#### 3.3.5. Hospital Level Percent Positive Scores and Correlations

Table 9 shows percent positive scores and Spearman correlations for the final SOPS Workplace Safety composite measures and items at the hospital level. The mean percent positive scores were based on the average of the hospital level composite measure scores. The percent positive scores for the composite measures ranged from 58% for *Addressing Workplace Aggression from Patients or Visitors* to 90% for *Protection from Workplace Hazards*. The mean percentage of respondents indicating symptoms of burnout on the *Work Stress/Burnout* item was 30%. The average percent positive score for *Overall Rating on Workplace Safety for Providers and Staff* was 53%.

Among the 10 SOPS Workplace Safety measures, 33 of the 45 correlations were statistically significant (*p* < 0.05). For the 6 composite measures, 10 of the 15 correlations were statistically significant (range, 0.39 to 0.79). *Workplace Aggression Policies, Procedures, and Training* was not significantly related to any other composite measures. *Work Stress/Burnout* was related to five of the other nine measures (range, −0.44 to −0.64). Overall Rating on Workplace Safety for Providers and Staff was significantly related to eight of the nine measures (range, 0.52 to 0.90).

## 4. Discussion

The Hospital Workplace Safety Supplemental Items demonstrated good psychometric properties. Internal consistency reliability estimates were acceptable for five of the six composite measures. At 0.67, the alpha for *Workplace Aggression Policies, Procedures, and Training* composite measure fell only slightly under the criterion of 0.70. All composite measures and all but two items had acceptable site-level reliability, indicating that the survey measures differentiated hospitals. Standardized factors loadings and model fit indices from the CFA provided support for the construct validity of the final six composite measures.

In addition, most composite measures were moderately, yet significantly, intercorrelated and also related to the *Overall Rating on Workplace Safety for Providers and Staff*, indicating adequate conceptual convergence among these measures. Only one composite measure, *Workplace Aggression Policies, Procedures, and Training*, was not associated with other composite measures or the overall rating. However, the small number of hospitals (*N* = 28) used in the analysis could have contributed to the non-significant findings. *Hospital Management Support for Workplace Safety* was strongly associated with the *Overall Rating on Workplace Safety for Providers and Staff* (*r_s_* = 0.90). Although this association is bivariate, and therefore unadjusted, it suggests the importance of support for workplace safety from hospital management on the overall rating of workplace safety.

Almost one-third of providers and staff across the study hospitals indicated experiencing at least one symptom of work stress/burnout. Furthermore, experiencing work stress/burnout was related to lower protection from workplace hazards and lower support for workplace safety from supervisors, managers, or clinical leaders and from hospital management. These could be important areas for future investigations, as burnout is a significant threat to the health and well-being of healthcare professionals [37].

While *Protection from Workplace Hazards* resulted in a high average percent positive score for the hospitals in our pilot study (90% positive), average percent positive scores for the remaining five composite measures were not as high, ranging from 58% to 82%. In addition, only about half of providers and staff rated their unit/work area as *Excellent* or *Very good* on workplace safety. Although the number of study hospitals was small and hospitals were not randomly selected, taken together, this evidence suggests ample room for improvement in multiple areas of workplace safety culture.

Two of the three items in the *Moving, Transferring, or Lifting Patients* composite measure had relatively high percentages of respondents answering, *Does not apply/Don’t know* (*DNA/DK*). Further analyses indicated that the percent of *DNA/DK* for these items was generally lower for staff expected to engage in moving, transferring, or lifting patients, such as those in clinical positions requiring direct patient care. Because staff seemed to be appropriately selecting *DNA/DK*, and given the importance of this aspect of workplace safety, we decided to retain this composite measure in the final item set.

### Limitations

The hospitals were not randomly selected and thus are not representative of all U.S. hospitals. However, the hospitals were selected using purposive sampling to vary by region, bed size, ownership, and hospital teaching status to ensure diversity on those key characteristics. In addition, respondents represented diverse positions within hospitals. These factors helped to assure that our findings are based on a fairly representative set of hospital respondents. In addition, while the supplemental items were added to the end of the SOPS Hospital Survey and made it longer, and our data collection occurred during the COVID-19 pandemic, we were able to obtain a sufficient overall response rate of 35%. In fact, the questions about workplace safety are likely to have been particularly salient to hospital providers and staff as a result of COVID-19-related concerns about PPE and stress/burnout.

## 5. Conclusions

The final Hospital Workplace Safety Supplemental Items released in October 2021 [38] extend the SOPS Hospital Survey by assessing additional dimensions of organizational culture that affect workplace safety for providers and staff. The supplemental item set consists of twenty-two supplemental items, including six composite measures, three single-item measures, one overall rating, and two background items. The final items and composite measures are not only psychometrically sound, but also measure different yet related aspects of workplace safety in hospitals. Healthcare organizations and researchers can use these items in conjunction with the SOPS Hospital Survey to assess how well the organizational culture in hospitals supports workplace safety and to identify strengths and areas for improvement. Given the importance of both workplace safety and patient safety in ensuring a comprehensive safety culture in healthcare [24], future work should more fully explore the relationship between patient safety culture and workplace safety culture.

## Figures and Tables

**Table 1:** Workplace safety supplemental items seven a priori composite measures.

Composite Measures	Description of Survey Item Content	Number of Survey Items
Protection from Workplace Hazards *	Procedures are in place to protect providers and staff from workplace hazards; providers and staff are provided with personal protective equipment (PPE), they are trained to use it and use it appropriately.	4
**Moving, Transferring, or Lifting Patients**	Equipment or assistive devices are available, staff use them when needed, and staff are available when needed to move, transfer, or lift patients.	3
Addressing Workplace Aggression from Patients or Visitors *	Physical and verbal aggression from patients or visitors is problematic; and effective policies, procedures, and training are in place to manage workplace aggression.	5
Addressing Workplace Aggression from Providers or Staff *	Physical and verbal aggression among providers and staff is problematic; and effective policies and procedures are in place to manage workplace aggression.	3
**Supervisor, Manager, or Clinical Leader Support for Workplace Safety**	Supervisors, managers, or clinical leaders monitor the workplace, seriously consider suggestions for improving safety, encourage providers and staff to report their concerns, and can be trusted to keep providers and staff safe.	4
**Hospital Management Support for Workplace Safety**	Hospital management shows that workplace safety is a top priority, provides adequate resources for workplace safety, and takes action to address concerns.	3
**Workplace Safety and Reporting**	Whether or not providers and staff notice or experience workplace safety hazards, injuries, or verbal or physical aggression and whether they report them; providers and staff can report concerns about workplace safety without fear of negative consequences.	9

* These are final measure names and differ slightly from the pilot test version.

**Table 2:** Criteria used to evaluate CFA model fit.

CFA Model Fit Criteria			
$\chi^{2}/df$	CFI	RMSEA	SRMR
<5.00	≥0.95	<0.06	<0.08

**Table 3:** Characteristics of pilot study hospitals (*N* = 28).

	Pilot Study Hospitals	
	Number	Percent
**Bed Size**		
6–24 beds	4	14%
25–49 beds	5	18%
50–99 beds	9	32%
100–199 beds	6	21%
200 beds or more	4	14%
**Teaching Status**		
Teaching	18	64%
Nonteaching	10	36%
**Hospital Ownership**		
Government (federal and non-federal)	11	39%
Non-government not for profit	11	39%
Investor owned (for profit)	6	21%
**U.S. Census Region**		
Northeast	7	25%
South	4	14%
Midwest	10	36%
West	7	25%

Note: Percentages may not add up to 100 percent due to rounding. U.S. states are categorized into regions, as follows (state abbreviations for each region are shown): Northeast: CT, MA, ME, NH, RI, VT, NJ, NY, PA; South: AL, AR, DC, DE, FL, GA, KY, LA, MD, MS, NC, OK, SC, TN, TX, VA, WV; Midwest: IA, IL, IN, KS, MI, MN, MO, ND, NE, OH, SD, WI; West: AK, AZ, CA, CO, HI, ID, MT, NM, OR, NV, UT, WA, WY.

**Table 4:** Characteristics of respondents in pilot hospitals.

	Respondents	
	Number	Percent
**Hospital Staff Position**		
Nursing Staff (RN, LVN, LPN, Nurse Practitioner)	2361	35%
Support Staff (Receptionist, Clerical Staff, Housekeeping Staff)	1301	20%
Other Clinical Staff (Pharmacist, Therapist, Technologist)	1196	18%
Other Position	847	13%
Supervisor, Manager, Clinical/Senior Leader	778	12%
Physician, Physician Assistant, Resident	162	2%
Total	6645	100%
Missing	39	-
Overall total	6684	-
**Unit/Work Area**		
Patient Care	1875	28%
Administration/Management	967	15%
Other Unit/Work Area	854	13%
Clinical Services	836	13%
Multiple Units/No Specific Unit	645	10%
Medical/Surgical	636	10%
Support Services	523	8%
Surgical Services	298	4%
Total	6634	100%
Missing	50	-
Overall total	6684	-

Note: Percentages may not add up to 100 percent due to rounding. RN = Registered Nurse; LVN = Licensed Vocational Nurse; LPN = Licensed Practical Nurse.

**Table 5:** Item analysis results (*N* = 6684).

Composite Measures and Items	% Positive	% MI	% DNA/DK
**Protection from Workplace Hazards (four items)**			
This unit has effective procedures to protect providers and staff from exposure to hazardous materials, contagious diseases, blood, or other bodily fluids	92%	<1%	12%
In this unit, providers and staff are provided with the appropriate personal protective equipment (PPE)	91%	<1%	10%
In this unit, providers and staff are trained to properly put on, use, and remove PPE	92%	1%	11%
In this unit, providers and staff use PPE appropriately	89%	1%	11%
**Moving, Transferring, or Lifting Patients (three items)**			
Equipment or assistive devices are available when needed to help move, transfer, or lift patients in this unit	75%	1%	42%
In this unit, staff use equipment or assistive devices when needed to help move, transfer, or lift patients, even if it takes more time	74%	1%	43%
In this unit, enough staff are available when needed to help move, transfer, or lift patients	64%	1%	39%
**Addressing Workplace Aggression from Patients or Visitors (five items)**			
In this unit, there is a problem with patients or visitors being physically aggressive toward providers or staff (negatively worded) *	59%	3%	25%
In this unit, there is a problem with patients or visitors being verbally aggressive toward providers or staff (negatively worded) *	45%	3%	24%
In this unit, there are effective policies and procedures to keep providers and staff safe from aggressive patients or visitors	71%	3%	22%
In this unit, providers and staff are trained to recognize early signs of aggressive behavior from patients or visitors	72%	3%	20%
In this unit, providers and staff are trained on how to deescalate or calm down aggressive behavior from patients or visitors	67%	3%	20%
**Addressing Workplace Aggression from Providers or Staff (three items)**			
In this unit, there is a problem with providers or staff being physically aggressive toward other providers or staff (negatively worded) *	91%	3%	14%
In this unit, there is a problem with providers or staff being verbally aggressive toward other providers or staff (negatively worded) *	74%	4%	13%
In this unit, there are effective policies and procedures to address providers and staff who behave aggressively toward other providers or staff	65%	4%	19%
**Supervisor, Manager, or Clinical Leader Support for Workplace Safety (four items)**			
My supervisor, manager, or clinical leader regularly monitors the workplace to identify unsafe working conditions for providers and staff	77%	2%	8%
My supervisor, manager, or clinical leader seriously considers provider or staff suggestions for improving workplace safety	78%	2%	6%
My supervisor, manager, or clinical leader encourages providers and staff to report their concerns about workplace safety	83%	2%	5%
My supervisor, manager, or clinical leader can be trusted to do the right thing to keep providers and staff safe	82%	2%	5%
**Hospital Management Support for Workplace Safety (three items)**			
The actions of hospital management show that the safety of providers and staff is a top priority	70%	2%	5%
Hospital management provides adequate resources to ensure the safety of providers and staff	70%	3%	5%
Hospital management takes action to address provider and staff concerns about workplace safety	69%	3%	6%
**Workplace Safety and Reporting (one item)**			
I can report my concerns about workplace safety without fear of negative consequences for me	76%	2%	2%
**Overall Rating on Workplace Safety for Providers and Staff (one item)**			
How would you rate your unit/work area on workplace safety for providers and staff?	50%	3%	N/A

Notes: % Positive = Strongly agree/Agree for positively worded items and Strongly disagree/Disagree for negatively worded items; MI = missing; DNA/DK = Does not apply or Don’t know. * % Positive are those who Strongly disagree/Disagree.

**Table 6:** Item analysis results: Work Stress/Burnout (*N* = 6508).

Work Stress/Burnout (one item)	%	% No Burnout and Burnout
Using your own definition of “burnout”, please select one of the answers below:		
I have no symptoms of burnout.	32%	69% (No symptoms of burnout)
I am under stress, and don’t always have as much energy as I did, but I don’t feel burned out.	37%	
I am definitely burning out and have one or more symptoms of burnout, e.g., emotional exhaustion.	21%	32% (Symptoms of burnout)
The symptoms of burnout that I am experiencing won’t go away. I think about work frustrations a lot.	8%	
I feel completely burned out. I am at the point where I may need to seek help.	3%	

Note: Percentages may not add to 100 percent due to rounding.

**Table 7:** CFA standardized factor loadings, final internal consistency reliability, and site-level reliability.

Measures and Items	CFA Standardized Factor Loading	Cronbach’s Alpha (Alpha If Item Deleted)	Site- Level Reliability
**Composite Measures and Items**			
**Protection from Workplace Hazards (three items)**	-	**0.87**	**0.80**
This unit has effective procedures to protect providers and staff from exposure to hazardous materials, contagious diseases, blood, or other bodily fluids	0.83	(0.81)	0.77
In this unit, providers and staff are provided with the appropriate personal protective equipment (PPE)	0.86	(0.78)	0.80
In this unit, providers and staff use PPE appropriately	0.78	(0.84)	0.74
**Moving, Transferring, or Lifting Patients (three items)**	-	**0.83**	**0.76**
Equipment or assistive devices are available when needed to help move, transfer, or lift patients in this unit	0.90	(0.70)	0.78
In this unit, staff use equipment or assistive devices when needed to help move, transfer, or lift patients, even if it takes more time	0.86	(0.71)	0.76
In this unit, enough staff are available when needed to help move, transfer, or lift patients	0.64	(0.88)	0.73
**Addressing Workplace Aggression from Patients or Visitors (two items)**	-	**0.89**	**0.86**
In this unit, there is a problem with patients or visitors being physically aggressive toward providers or staff (negatively worded)	0.85	(N/A)	0.87
In this unit, there is a problem with patients or visitors being verbally aggressive toward providers or staff (negatively worded)	0.94	(N/A)	0.83
**Workplace Aggression Policies, Procedures, and Training (two items)**	-	**0.67**	**0.80**
In this unit, there are effective policies and procedures to keep providers and staff safe from aggressive patients or visitors	0.84	(N/A)	0.69
In this unit, providers and staff are trained on how to deescalate or calm down aggressive behavior from patients or visitors	0.59	(N/A)	0.86
**Supervisor, Manager, or Clinical Leader Support for Workplace Safety (three items)**	-	**0.92**	**0.77**
My supervisor, manager, or clinical leader regularly monitors the workplace to identify unsafe working conditions for providers and staff	0.85	(0.91)	0.72
My supervisor, manager, or clinical leader encourages providers and staff to report their concerns about workplace safety	0.89	(0.88)	0.75
My supervisor, manager, or clinical leader can be trusted to do the right thing to keep providers and staff safe	0.92	(0.87)	0.78
**Hospital Management Support for Workplace Safety** **(three items)**	-	**0.96**	**0.85**
The actions of hospital management show that the safety of providers and staff is a top priority	0.94	(0.95)	0.84
Hospital management provides adequate resources to ensure the safety of providers and staff	0.95	(0.95)	0.85
Hospital management takes action to address provider and staff concerns about workplace safety	0.95	(0.95)	0.84
**Single-Item Measures**			
**Addressing Verbal Aggression from Providers or Staff**			
In this unit, there is a problem with providers or staff being verbally aggressive toward other providers or staff	-	-	0.80
**Workplace Safety and Reporting**			
I can report my concerns about workplace safety without fear of negative consequences for me	-	-	0.76
**Work Stress/Burnout**			
Using your own definition of “burnout”, please select one of the answers below	-	-	0.62
**Overall Rating**			
How would you rate your unit/work area on workplace safety for providers and staff?	-	-	0.86

Notes: Composite measure scores at the respondent level were calculated as means of their respective constituent items. Survey measures are shown in the order they appear in the survey within each category: composite measures, single-item measures, and overall rating. All factor loadings were statistically significant (*p* < 0.05).

**Table 8:** Confirmatory factor analysis: Model fit indices.

CFA Model Fit Indices					
$\chi^{2}\;$	df	$\chi^{2}/df$	CFI	RMSEA (CI)	SRMR
1202.24 *	89	13.5	0.98	0.04 (0.04–0.05)	0.05

* Chi-square was statistically significant (*p* < 0.05). CI = 90% confidence intervals. CFI = Comparative Fit Index. RMSEA = Root Mean Square Error of Approximation. SRMR = Standardized Root Mean Square Residual.

**Table 9:** Hospital-level percent positive scores and correlations for the final SOPS Workplace Safety measures (*N* = 28).

Workplace Safety Measures	Mean Score	SD	Correlations								
			(1)	(2)	(3)	(4)	(5)	(6)	(7)	(8)	(9)
**Composite Measures**	**% Positive**										
(1) Protection from Workplace Hazards	90%	4%	-								
(2) Moving, Transferring, or Lifting Patients	73%	10%	**0.57**	-							
(3) Addressing Workplace Aggression from Patients or Visitors	58%	13%	**0.39**	**0.58**	-						
(4) Workplace Aggression Policies, Procedures, and Training	69%	11%	−0.14	0.20	0.02	-					
(5) Supervisor, Manager, or Clinical Leader Support for Workplace Safety	82%	7%	**0.55**	**0.60**	**0.53**	0.15	-				
(6) Hospital Management Support for Workplace Safety	70%	10%	**0.79**	**0.76**	**0.49**	0.20	**0.73**	-			
**Single-Item Measures**	**% Positive**										
(7) Addressing Verbal Aggression from Providers or Staff	78%	9%	0.27	**0.57**	**0.60**	**0.41**	**0.72**	**0.48**	-		
(8) Workplace Safety and Reporting	78%	8%	**0.44**	**0.47**	**0.55**	0.30	**0.80**	**0.72**	**0.72**	-	
	**% Burned out**										
(9) Work Stress/Burnout	30%	8%	**−0.61**	−0.32	−0.15	0.20	**−0.64**	**−0.60**	−0.32	**−0.44**	-
**Overall Rating**	**% Positive**										
(10) Overall Rating on Workplace Safety for Providers and Staff	53%	11%	**0.75**	**0.79**	**0.52**	0.13	**0.77**	**0.90**	**0.53**	**0.68**	**−0.64**

Note: SD = standard deviation. All correlations shown in bold are statistically significant (*p* < 0.05).

## Data Availability

Some of the de-identified Workplace Safety Supplemental Item data are available upon request for research purposes.

## Supplementary Materials

The following supporting information can be downloaded at: https://www.mdpi.com/article/10.3390/ijerph19116815/s1, Table S1: Pilot survey items dropped from final item set based on Technical Expert Panel (TEP) review.
