# Supplementary material for: Development, Pilot Study, and Psychometric Analysis of the AHRQ Surveys on Patient Safety Culture™ (SOPS®) Workplace Safety Supplemental Items for Hospitals
Source: Int J Environ Res Public Health. 2022 Jun 2;19(11):6815. doi: 10.3390/ijerph19116815 (PMC9179961; doi:10.3390/ijerph19116815)
Supplement: Supplementary file 1 [file ijerph-19-06815-s001.zip › ijerph-1646205-Supplementary.pdf]

Table S1. Pilot survey items dropped from final item set based on Technical Expert Panel (TEP) review.

Item(s) dropped	Reasons for dropping
**Protection From Workplace Hazards**	
In this unit, providers and staff are trained to properly put on, use, and remove PPE	To shorten the survey, the TEP recommended dropping this item because it was not necessary to have three items about PPE. Also, this item was 92% positive.
**Addressing Workplace Aggression from Patients or Visitors**	
In this unit, providers and staff are trained to recognize early signs of aggressive behavior from patients or visitors	To shorten the survey, the TEP recommended dropping this item because the content was covered by another, more inclusive item in this composite measure.
**Addressing Workplace Aggression from Providers or Staff**	
In this unit, there is a problem with providers or staff being physically aggressive toward other providers or staff	To shorten the survey, the TEP recommended dropping this item because physical aggression among providers and staff is rare, so a question focusing on this topic did not seem necessary.
In this unit, there are effective policies and procedures to address providers and staff who behave aggressively toward other providers or staff	To shorten the survey, the TEP recommended dropping this item because hospitals most likely have human resource policies on how to address aggressive behavior from providers and staff. Therefore, this item did not seem necessary.
**Supervisor, Manager, or Clinical Leader Support for Workplace Safety**	
My supervisor, manager, or clinical leader seriously considers provider or staff suggestions for improving workplace safety	To shorten the survey, the TEP recommended dropping this item because staff have no real way of knowing if an idea was considered seriously. Additionally, staff could have offered unacceptable suggestions which could not be implemented. Finally, seriously considering suggestions may not mean that ultimately an action have been taken.

Notes: Response options were Strongly disagree, Disagree, Neither agree nor disagree, Agree, Strongly agree, Does not apply or Don’t know. TEP = Technical Expert Panel.
